# The project for objective measures using computational psychiatry technology (PROMPT): Rationale, design, and methodology

**DOI:** 10.1016/j.conctc.2020.100649

**Published:** 2020-08-18

**Authors:** Taishiro Kishimoto, Akihiro Takamiya, Kuo-ching Liang, Kei Funaki, Takanori Fujita, Momoko Kitazawa, Michitaka Yoshimura, Yuki Tazawa, Toshiro Horigome, Yoko Eguchi, Toshiaki Kikuchi, Masayuki Tomita, Shogyoku Bun, Junichi Murakami, Brian Sumali, Tifani Warnita, Aiko Kishi, Mizuki Yotsui, Hiroyoshi Toyoshiba, Yasue Mitsukura, Koichi Shinoda, Yasubumi Sakakibara, Masaru Mimura, PROMPT collaborators

**Affiliations:** a Department of Neuropsychiatry, Keio University School of Medicine, 35 Shinanomachi, Shinjuku, Tokyo, 160-8582, Japan; b Department of Health Policy and Management, Keio University, 35 Shinanomachi, Shinjuku, Tokyo, 160-8582, Japan; c Oizumi Hospital, 6-9-1 Oizumi-gakuencho, Nerimaku, Tokyo, 178-0061, Japan; d Sato Hospital, 948-1 Kunugutsuka, Nanyo, Yamagata, 999-2221, Japan; e Biwako Hospital, 1-8-5 Sakamoto, Otsu, Shiga, 520-0113, Japan; f Department of System Design Engineering, Keio University, 3-14-1 Hiyoshi, Minato-kita, Yokohama, Kanagawa, 223-0061, Japan; g Department of Computer Science, School of Computing, Tokyo Institute of Technology, 4259 Nagatsuda, Yokohama, Kanagawa, 226-8503, Japan; h Department of Biosciences and Informatics, Keio University, 3-14-1 Hiyoshi, Minato-kita, Yokohama, Kanagawa, 223-0061, Japan; i Center for Systems Medicine, Keio University, 35 Shinanomachi, Shinjuku, Tokyo, 160-8582, Japan; j FRONTEO, Inc., 2-12-23 Minato-Minami, Minato, Tokyo, 108-0075, Japan

**Keywords:** Depression, Neurocognitive disorder, Machine learning, Screening, Natural language processing, PROMPT, Project for Objective Measures Using Computational Psychiatry Technology, UMIN, University Hospital Medical Information Network, MCI, mild cognitive impairment, AMED, Japan Agency for Medical Research and Development, DSM-5, Diagnostic and Statistical Manual of Mental Disorders, Fifth Edition, M.I.N.I., Mini-International Neuropsychiatric Interview, RGB, red, green, blue, UV, ultraviolet, SCID, Structural Clinical Interview for DSM-5, ISO, International Organization for Standardization, FedRAMP, Federal Risk and Authorization Management Program, IEC, International Electrotechnical Commission, HAM-D, Hamilton Depression Rating Scale, MADRS, Montgomery-Asberg Depression Rating Scale, BDI-II, Beck Depression Inventory, Second Edition, F0, fundamental frequency, F1, F2, F3, first, second, and third formant frequencies, CPP, cepstral peak prominence, MFCC, mel-frequency cepstrum coefficients, SVR, Support Vector Regression, SVM, Support Vector Machine, RF, Random Forest, Adaboost, Adaptive Boosting, Adabag, Adaptive Bagging, CNN, Convolutional Neural Networks, GCNN, Gated Convolutional Neural Networks, BNN, Bayesian Neural Networks, LSTM, Long Short-Term Memory Networks, MDD, Major depressive disorder, UI, uncertainty interval, YLDs, years lived with disability, PET, positron emission tomography, MRI, magnetic resonance imaging, MARS, Motor Agitation and Retardation Scale, MMSE, Mini-Mental State Examination, MoCA, Montreal Cognitive Assessment, BD, Bipolar disorder, YMRS, Young Mania Rating Scale, PSQI, Pittsburgh Sleep Quality Index, CDR, Clinical Dementia Rating, LM, Wechsler Memory Scale-Revised Logical Memory, CDT, Clock Drawing Test, NPI, Neuropsychiatric Inventory, GDS, Geriatric Depression Scale

## Abstract

**Introduction:**

Depressive and neurocognitive disorders are debilitating conditions that account for the leading causes of years lived with disability worldwide. However, there are no biomarkers that are objective or easy-to-obtain in daily clinical practice, which leads to difficulties in assessing treatment response and developing new drugs. New technology allows quantification of features that clinicians perceive as reflective of disorder severity, such as facial expressions, phonic/speech information, body motion, daily activity, and sleep.

**Methods:**

Major depressive disorder, bipolar disorder, and major and minor neurocognitive disorders as well as healthy controls are recruited for the study. A psychiatrist/psychologist conducts conversational 10-min interviews with participants ≤10 times within up to five years of follow-up. Interviews are recorded using RGB and infrared cameras, and an array microphone. As an option, participants are asked to wear wrist-band type devices during the observational period. Various software is used to process the raw video, voice, infrared, and wearable device data. A machine learning approach is used to predict the presence of symptoms, severity, and the improvement/deterioration of symptoms.

**Discussion:**

The overall goal of this proposed study, the Project for Objective Measures Using Computational Psychiatry Technology (PROMPT), is to develop objective, noninvasive, and easy-to-use biomarkers for assessing the severity of depressive and neurocognitive disorders in the hopes of guiding decision-making in clinical settings as well as reducing the risk of clinical trial failure. Challenges may include the large variability of samples, which makes it difficult to extract the features that commonly reflect disorder severity.

**Trial Registration:**

UMIN000021396, University Hospital Medical Information Network (UMIN).

## 1 Introduction

Depressive disorders and neurocognitive disorders are common, disabling, and debilitating psychiatric conditions. However, these disorders are difficult to diagnosis objectively. Currently the most popular severity measurement tools for depression are subjective evaluations, and there are, so far, no known objective biomarkers that are reliable and easy-to-use in clinical settings. Dementia and its intermediate stage known as mild cognitive impairment (MCI), is increasingly affecting people as the global population ages. The biological mechanisms of dementia may be better understood than those of depression, and several early diagnostic methods are already possible [[1], [2], [3], [4]]. However, similar to the case of depression, there are no reliable biomarkers for dementia, and rating scales used to test cognitive function may place unnecessary burdens on the subject and can be influenced by the subject's education level.

Due to these factors, there are limits to the “gold standard” rating scales used in clinical settings and trials, and there are no ideal biomarkers for depressive and neurocognitive disorders. But at the same time, psychiatrists are able to infer a certain amount about a patient's severity by the way they act in clinical settings; for example, how the patient enters the room, sits in a chair, or speaks to the interviewer. In this way, psychiatrists can observe the patient's condition and determine if their treatment is effective. But those observations are difficult to quantify for practical application.

With recent developments in many technological fields, the collection and analysis of a variety of data sets has become easier and less expensive by using specialized electronic devices, and quantification of previously subjective data is increasingly possible [[5], [6], [7], [8]]. In many cases, studies that collect large amounts of data from electronic devices also use machine learning to estimate the presence and/or severity of illnesses. When applied to this goal, machine learning approaches are valuable, as data from such applications often contain complex cross-sectional and longitudinal patterns. By collecting such data with diagnoses and/or severity information as labels, we can develop novel machine learning techniques to discover these complex patterns, which can in turn provide objective indices and predictive models for diagnosis (categorical classification) and severity assessment (continuous variable prediction), as well as for judging whether there has been an improvement/deterioration in a patient's condition since their previous visit (categorical classification). Through these machine learning tasks, it is also possible to gain additional insights into which clinical characteristics are helpful in diagnosing and evaluating severity, how to identify characteristics that parallel symptom improvement, and more.

The Project for Objective Measures Using Computational Psychiatry Technology (PROMPT), which is funded by the Japan Agency for Medical Research and Development (AMED), is an industry-academia collaborative research project that aims to develop new techniques for diagnosing and evaluating illness severity utilizing the technology described above, with the hope that this research will prove useful in every-day clinical settings and trials.

## 2 Methods

### 2.1 Participants

This study is a multi-site prospective observational study. Participants are recruited at seven hospitals and three outpatient clinics that specialize in treating either mood disorders or dementia, or both, in five different prefectures in Japan. Patient recruitment is conducted in the following locations and hospitals: Tokyo (Keio University Hospital, Tsurugaoka Garden Hospital, Oizumi Hospital, Komagino Hospital); Shiga (Biwako Hospital); Yamagata (Sato Hospital); Fukushima (Asaka Hospital). Outpatient clinics were used for additional patient recruitment in Tokyo (Oizumi Mental Clinic, Asakadai Mental Clinic) and Kanagawa (Nagatsuda Ikoinomori Clinic). Healthy controls are recruited through an advertisement on our research group website or through Silver Human Resource Centers (employment/volunteer centers for seniors). Participants are inpatients or outpatients aged ≥20 years, who met the Diagnostic and Statistical Manual of Mental Disorders, Fifth Edition (DSM-5) criteria for major depressive disorder, bipolar disorder, major neurocognitive disorder, and mild neurocognitive disorder. Patients with subjective cognitive impairment (i.e., patients who feel they are cognitively impaired, but when tested, are not shown to have abnormalities) are also included in this study. Exclusion criteria include: (1) paralysis or involuntary movement in the face or body; and (2) inability to speak (e.g., removal of vocal cords). Healthy controls are screened by using the Mini-International Neuropsychiatric Interview (M.I.N.I.) and MMSE, and are excluded if they have a history of psychiatric disorders or show cognitive impairment. Researchers obtain written informed consent from all participants. In cases where patients are judged to be decisionally impaired, the patients’ guardians will give consent. Participants are able to leave the study at any time.

### 2.2 Assessments

All assessments are undertaken by trained research psychiatrists and/or psychologists. Raters are required to take a 40-h training session, comprised of a 20-h educational module and a 20-h supervision portion. Moreover, raters' assessments will be randomly checked by other raters using the recorded videos and voice data to keep inter-rater reliability high. Clinical characteristics (e.g., age, sex, duration of illness), past medical history, and currently prescribed medications are collected using patients' medical charts. RGB and infrared video recordings [RealSense R200 (Intel Corporation)/Microsoft Kinect for Windows v2 (Microsoft Corporation)], and voice recordings using an array microphone [Classis RM30W (Beyerdynamic GmbH & Co. KG)/PRO8HEx Hypercardioid Dynamic Headworn Microphone (Audio-Technica Corporation)], are captured during a 10-min interview with a psychiatrist and/or psychologist. During the interview, conversations between the interviewer and patient cover topics that arise in normal clinical practice, such as mood, daily living, sleep, events in the past week, concerns, etc. After the 10-min clinical interview, a semi-structured interview using the clinical assessment tools is conducted (Table 1). In addition to participating in the above-mentioned interview recordings, participants are asked to wear wearable devices [Silmee W20 (TDK Corporation)] until their next assessment. Silmee is a wristband-type wearable monitor equipped with an accelerometer, gyrometer, pulse sensor, thermometer, and UV meter. We make the use of wearable devices optional, as it is possible that some participants will see it as a burden. In order to collect various data from the same patients in different states, assessments are done up to 10 times for each patient during the study period. Visit intervals are not fixed, but we attempt to time them for when patients' clinical symptoms have changed from the last visit (e.g., if we learn from the treating psychiatrist that a patient has recovered, we attempt to see the patient at that time), so that we can input datasets reflecting various illness severities into the machine learning program. The minimum interval sets are one week for patients with depression and one month for healthy volunteers. The Structural Clinical Interview for DSM-5 (SCID) is performed to the greatest degree feasible to confirm the diagnoses during the follow up period. Normal treatment is continued during the study period. The documents pertaining to this research are only stored in cabinets that lock within a research room of the Keio University School of Medicine's Department of Neuropsychiatry. We assign research numbers to data that will be used in the study, and from there on, the data are managed using those numbers. Once numbers are assigned, all data are completely separated from any personal identifiers. Additionally, case report forms are managed using electronic data capture.

**Table 1:** A semi-structured interview using clinical assessment tools.

Type of assessment	Time administered	Healthy controls	MDD	BD	Neurocognitive disorder
HAM-D	Every visit	✓	✓	✓	
MADRS	Every visit	✓	✓	✓	
YMRS	Every visit	✓	✓	✓	
BDI-II	Every visit	✓	✓	✓	
PSQI	Every visit	✓	✓	✓	
MMSE	Screening, every visit	✓			✓
CDR	Every visit	✓			✓
LM (Immediate/Delayed)	Every visit	✓			✓
CDT (copying/free-drawing)	Every visit	✓			✓
NPI	Every visit	✓			✓
GDS	Every visit	✓			✓
M.I.N.I.	Screening	✓			✓
SCID	Once during the follow up	✓	✓	✓	✓

MDD: Major depressive disorder, BD: Bipolar disorder, HAM-D: Hamilton Depression Rating Scale, MADRS: Montgomery-Asberg Depression Rating Scale, YMRS: Young Mania Rating Scale, BDI-II: Beck Depression Inventory Second Edition, PSQI: Pittsburgh Sleep Quality Index, MMSE: Mini-Mental State Examination, CDR: Clinical Dementia Rating, LM: Wechsler Memory Scale-Revised Logical Memory, CDT: Clock Drawing Test, NPI: Neuropsychiatric Inventory, GDS: Geriatric Depression Scale, M.I.N.I.: Mini-International Neuropsychiatric Interview, SCID: Structural Clinical Interview for DSM-5.

All these data are stored securely in Microsoft Azure. Microsoft Azure is a highly reliable cloud-based system, and it has wide compliance with industry-specific and global regulations, such as: adherence to ISO 27001, an international regulation for information security management systems; adherence to FedRAMP, a cloud-computing security standard in the United States; and adherence to ISO/IEC 27018, the international performance standard for regulating how personal information is handled by cloud service providers.

### 2.3 Analysis

The machine learning models for PROMPT are trained to perform the following tasks: 1) predict whether a subject has or does not have depression/neurocognitive disorders for screening purposes; 2) predict the severity of a subject's depression/cognitive decline based on results from severity rating scales such as the Hamilton Depression Rating Scale (HAM-D) (including the 21, 17, and 6 item versions' scores) [9], Montgomery-Asberg Depression Rating Scale (MADRS) [10], Beck Depression Inventory, Second Edition (BDI-II), and MMSE with a known margin of error for the predicted rating; 3) predict the improvement or deterioration of a subject's depressive state/cognitive function with respect to a previously recorded state if the subject has had a prior assessment by the system; and 4) predict the scores of individual items in a depression/cognitive rating scale that are indicators of different aspects of a subject's depression/cognitive states, such as depressed mood, anhedonia, insomnia, anxiety, and psychomotor retardation/agitation for depression, or orientation to time and place, memory, attention and calculation, language, and visuospatial perception for neurocognitive disorders.

The data used to train these machine learning models are multimodal in nature, including facial expression and eye blinking features extracted from RGB video recordings, body motion features extracted from infrared recordings, and voice features extracted from audio recordings. We first perform data cleaning and feature engineering to construct feature vectors in which the machine learning algorithms can more easily find patterns that can correctly identify healthy and depressed subjects or predict a fine gradient of depression severity from a subject's physical symptoms.

### 2.4 Extracted data

In audio engineering, phonic data are often used to describe the sound generation from the vocal cord and sound modulation from the shape of the mouth and the position of the tongue. To use these physical properties in our machine learning models, we extract phonic data from audio recordings with software such as Praat [11] and openSMILE [12] at 10-ms intervals. These phonic data include: fundamental frequency (F0); first, second, and third formant frequencies (F1, F2, F3); cepstral peak prominence (CPP); and mel-frequency cepstrum coefficients (MFCC).

To discover patterns at a higher level, prosodic speech data are extracted from audio recordings, including: rate of speech, which measures the number of syllables spoken per minute; delay of reply, which measures the length of delay between the end of the physician's sentence and the beginning of the subject's subsequent sentence; and pause time, which measures the length of delay between two consecutive sentences spoken by the subject.

Facial features are extracted from video recordings with software such as OKAO Vision and Openface [13,14]. The data extracted include predicted facial expressions of the subject in each frame of the video recording, and the inverse distance between the upper and lower eye lids.

Regarding body motion, the speed statistics and angles formed by four joints in XYZ dimension, namely Spine Shoulder, Head, Shoulder Right, and Shoulder Left, are utilized as features. These joints are extracted either by Kinect V2 joint map, or from Intel RealSense.

We collect daily activity data for the subjects using wearable devices as described above. Daily activity data targeted for collection include number of steps taken, energy expended, body motion, sleep state, skin temperature, heart rate, and UV exposure index.

### 2.5 Feature engineering

For some machine learning models, we need to perform feature engineering to summarize the time-course data extracted from the raw audio and video recordings, and to capture the relationship between pairs of time-course data. The following feature engineering approaches are used to construct features from the multi-modal data as input to the machine learning models for predicting a subject's depression/cognitive status and/or severity using the following methods: 1) space-delay matrix [15] that computes all pair-wise similarities between the extracted data (space) at each delay from a set of different delay scales (delay); 2) distribution statistics (5-, 25-, 50-, 75-, 95-quantile and mean and standard deviation); 3) Markov transition probabilities for the state change between two adjacent time-series samples; 4) similarity measures between different data; and 5) decision-tree-based quantization of data.

### 2.6 Machine learning architecture

We take two approaches to the machine learning architecture: one based on non-deep-learning machine learning algorithms, utilizing feature selection of the engineered features and meta-models; and one based on deep-learning algorithms.

For the non-deep-learning-based machine learning architecture, we first perform feature selection to choose a subset of the engineered features to build our models. The parameters obtained through feature engineering are passed to an elastic-net model [16] for feature selection. The labels of the dependent variables are regressed on the feature vector and an elastic-net model is fitted. The fitted model has a sparse set of coefficients; i.e., many of the features’ coefficients will be forced to zero during fitting and contribute nothing to the prediction of the labels. The features in the feature vector that have non-zero coefficients are retained as selected features and used to build the next layer of the machine learning algorithm.

Next, the selected features from the elastic-net feature selection layer are used to train the first layer models of the meta-model. Models used in the second layer include algorithms such as Support Vector Regression (SVR) [17], Support Vector Machine (SVM) [18], XGBoost [19], Random Forest (RF) [20], Adaptive Boosting (Adaboost) [21], and Adaptive Bagging (Adabag) [22]. The same selected features (features with non-zero coefficients) are used in each of the machine learning models and the labels predicted by each model are passed as features to the second layer of the meta-model.

For the second layer, we can use an algorithm with logistic regression or SVM for classification, or one with a linear model or SVR for regression. The features for this layer are the predicted labels from the previous machine learning layer, and the true labels are regressed against these predicted labels to train the machine learning model.

For deep-learning-based models, we use deep-learning architectures such as Convolutional Neural Networks (CNN) [23,24], Gated Convolutional Neural Networks (GCNN) [25], Bayesian Neural Networks (BNN) [26], and Long Short-Term Memory Networks (LSTM) [27]. For these models, the time-course features extracted from the raw video and audio data are used directly as input, instead of the engineered features. It should be noted that for either deep-learning or non-deep-learning-based architectures, the models are not limited to those listed above.

For the improvement/deterioration model we use the non-deep-learning machine learning models, where each input feature vector is constructed from the data of two separate interviews with the same subject. For each of the interviews with the same subject, the feature vector is constructed as described above. To construct the feature vector for the improvement/deterioration model, the feature vector of the prior interview is divided elementwise by the feature vector of the latter interview. This new vector of element-wise ratios of the feature vectors of the two interviews is used as the feature vector for the improvement/deterioration model. The machine learning architecture for the improvement/deterioration model is the same as the model presented above.

### 2.7 Sample size

To predict the sample size required for the supervised learning performances, we use learning curves to estimate the number of samples required to reach 90% accuracy for classification tasks. An inverse exponential model is fitted to pairs of sample size and cross-validation accuracy to predict the number of samples necessary. For depression, based on the preliminary data that we collected, we estimated a need for approximately 200 patients and 100 healthy volunteers; for dementia, we estimated a need for 100 patients and 100 healthy volunteers. Assuming an average of three assessments per individual participant, we therefore set a target of 1500 datasets from 500 participants.

## 3 Discussion

The PROMPT study is unique in its purpose and integrative approach. The main purpose of PROMPT is to develop objective digital biomarkers for the assessment of depression/neurocognitive disorders in the hopes of guiding clinical decision-making in clinical settings. There will be tremendous value in noninvasive and easy-to-use methods that do not put additional burdens on clinical practice, and which can be repeatedly conducted not only in daily clinical settings, but also in clinical trials.

Currently, depressive and neurocognitive disorders are debilitating conditions that account for the leading causes of years lived with disability worldwide. Major depressive disorder (MDD) affects approximately 6% of the adult population worldwide each year [28], and the prevalence in 2017 is estimated to have been 264.5 million people [95% uncertainty interval (UI) 246.3 to 286.3]. Moreover, depressive disorder is the third leading cause of years lived with disability (YLDs) that contributes to 43.1 million YLDs (95%UI 30.5 to 58.9) [29]. Pharmacotherapy is one of the mainstays of depression treatment, and many efforts to develop new antidepressant treatments have been made. However, clinical trials for antidepressant medications face tremendous difficulties. The reasons for these difficulties include multiple factors, such as: 1) the mechanisms of an illness are not fully understood; 2) the heterogeneity of the targeted population; 3) difficulty in recruiting patients with severe symptoms; 4) too many placebo responders; and so on. Poor reliability of measurement, poor interview quality, and rater bias are also important factors that contribute to a number of these reasons for trial failure [30,31].

Until now, options for assessing and diagnosing patients with depressive and neurocognitive disorders have been overly-subjective, or have utilized unreliable biomarkers. For depressive disorders, the most popular severity measurement tools include the Hamilton Depression Rating Scale (HAM-D) and Montgomery-Asberg Depression Rating Scale (MADRS). Although HAM-D and MADRS are clinician-rated assessment tools, these measures mainly depend on subjective reports by the patients. Such rating scales can be influenced by the patient's personality and/or the interviewer's ability/skill. It is also common for the anchor point to be ambiguous, among other issues. Several other biological, objective methods have been investigated with the aim of ensuring a more objective measurement of depression severity, such as monoamine levels in cerebrospinal fluids [32], cytokines [33], positron emission tomography (PET) [34], neuroendocrine tests [35], and magnetic resonance imaging (MRI) [36]. In contrast, digital biomarkers can be applied as noninvasive and easy-to-use biomarkers in clinical settings, and they can be used during a treatment course repeatedly.

In terms of depression, the various domains of human expression, such as facial movements, speech, and motor movements, have been identified as observable features in depressed patients since Hippocrates's era [37]. Several studies linked depression with less eye contact, overall sluggishness, slumping back posture, etc. [[38], [39], [40], [41]]. These observable psychomotor abnormalities continue to be regarded by experts as essential and critical features of depression, especially melancholic depression or melancholia [[42], [43], [44]]. Specifically, observable signs of patients, such as facial expression and speech rate, are important characteristics of depressive disorders, but psychomotor disturbances in particular are considered one of the most fundamental features of depression, especially melancholic depression [43]. They are also one of the diagnostic symptoms of major depressive episodes and manic episodes [45]. Psychomotor disturbances may have predictive value for antidepressant treatments, especially for electroconvulsive therapy [40]. Some rating scales have been developed for psychomotor disturbances, including the CORE measurement [46] and the Motor Agitation and Retardation Scale (MARS) [47]. However, these measurements rely on the subjective judgment of the clinicians, and no reliable and/or validated objective measurement methods for psychomotor disturbances have been developed. Therefore, PROMPT strives to overcome these historical issues. In addition, our model could be used as an assessment tool for psychomotor disturbances, and for distinguishing melancholic depression from heterogeneous DSM-defined major depression. It could also be used for investigating the underlying neurobiology of psychomotor disturbances in collaboration with neuroimaging/neurophysiological measurements in future studies. Moreover, in clinical settings, clinicians usually assess depressive symptoms as typical or atypical, and consider the possibility of a bipolar depressive episode or the possibility of a depressive state due to other medical conditions, such as thyroid dysfunction. By combining this clinical information with acquired digital data, our developed digital biomarkers may be used to detect depressive subtypes or depressive state due to such medical comorbidities.

In addition to depression, the number of individuals who live with neurocognitive disorders world-wide is estimated to be 45 million (95%UI 39.7 to 50.4) [29], and these disorders contribute to 6.5 million YLDs (95%UI 4.7 to 8.6). Furthermore, neurocognitive disorders are the fifth leading cause of death globally, accounting for 2.4 million (95% UI 2.1 to 2.8) deaths [48]. It is believed that in the future, this number may increase to up to 82 million by 2030, and 152 million by 2050 [49]. Additionally, mild cognitive impairment (MCI), which is an intermediate stage between the expected cognitive decline of normal aging and the decline caused by a neurocognitive disorder, has an estimated prevalence of 10%–20% in individuals aged ≥65 years [50]. The importance of early intervention and prevention of disease through the modification of therapy methods is being emphasized more and more; however, examinations such as amyloid PET or cerebrospinal fluid tests are not practical in terms of the invasiveness and cost (e.g., 2000 USD for amyloid PET in Japan as of 2020), as well as the facility equipment requirements. In addition, although there are several rating scales used to test cognitive function, such as the Mini-Mental State Examination (MMSE) [51] and Montreal Cognitive Assessment (MoCA) [52], the calculation and memorization components of these evaluations may place unnecessary burdens on the subject and can be influenced by the subject's education level. Also, when performing cognitive assessments at the preclinical stage, it is difficult to distinguish between disease-related changes and changes caused by normal aging, since cognitive impairment is still comparatively minor at that stage. Learning effects can also be a significant problem when a patient is assessed repeatedly, especially in the early phase of a disorder. This is because patients with slight cognitive impairment may end up memorizing the testing procedures, which would defeat the purpose of the exams. On the other hand, similar to facial expression and psychomotor disturbances in depression patients, clinicians can gain information on dementia patients from instances when they hesitate in their speech trying to recall a word, or when they try to gloss over the fact they cannot remember something. As dementia symptoms progress, patients lose their motivation, as well as interest in things around them, and these effects are reflected in the patients' speech and facial expressions. But these observations are still subjective; it would be highly beneficial if a new approach is developed that can identify high risk patients in a quantifiable manner.

Challenges of this study are as follows. First, the large variability of the subjects makes it difficult to extract the features that commonly reflect disorder severity. For example, if we learn that one's conversational response time is slower than a healthy control's, we still do not know if he/she has psychomotor retardation, because we do not know his/her original speed of speech. But at the same time, psychiatrists can judge if someone has psychomotor retardation even if they do not know what he/she was like before the onset of illness. Psychiatrists most likely gather multimodal information from patients for a comprehensive judgement, and a machine may be able to do the same, as long as it is given the same modalities. Nevertheless, the variability of the samples is the most concerning matter for this study, and though this could be resolved to a certain degree by gathering a larger number of datasets, we may still see the machine learning models' accuracy hit a ceiling at some point. Second, recruiting severe patients is difficult. As this study does not focus on intervention, recruitment may not be as large a problem in this case, but recruiting severe patients is an inherent difficulty in clinical studies. Imbalanced samples for different severities caused by recruitment difficulties may prohibit the machine learning algorithms from achieving a high prediction accuracy. Third, it is very important to keep inter-rater reliability high when diagnosing and/or assessing patients, as assessment scale scores will be the labels for machine learning. Anticipating this issue, the study team developed educational modules to maintain a high quality of ratings, and the inter-rater reliability will be tested using random sampling during the study period. Finally, since data will be collected from typical clinical settings, the recordings may contain a significant amount of optical and acoustic noise from the background, or due to inconsistent instrument setup. Well-designed preprocessing and data cleaning steps will be important to provide high quality features for the machine learning algorithms.

## Ethics approval and consent to participate

This study was approved by the Institutional Review Board of Keio University School of Medicine and the participating medical facilities. Researchers obtain written informed consent from all participants. In cases where patients were judged to be decisionally impaired, the patients’ guardians will give consent. Participants are able to leave the study at any time.

## Preprint

This article has been posted on preprint site: https://doi.org/10.1101/19013011.

## Funding

This research is supported by the 10.13039/100009619Japan Agency for Medical Research and Development (AMED) under Grant Number JP18he1102004. The Grant was awarded on Oct. 29, 2015 and ends on Mar. 31, 2019. The funding source did not participate in the design of this study and will not have any hand in the study's execution, analyses, or submission of results.

Japan Agency for Medical Research and Development (AMED) 20F Yomiuri Shimbun Bldg. 1-7-1 Otemachi, Chiyoda-ku, Tokyo 100-0004 Japan Tel: +81-3-6870-2200, Fax: +81-3-6870-2241, Email: jimu-ask@amed.go.jp.

## Authors’ contributions

T. Kishimoto, AT, KL, KF, TF, MK, M. Yoshimura, YT, TH, YE, T. Kikuchi, MT, SB, JM, BS, TW, AK, M. Yotsui, HT, YM, KS, YS, MM contributed to the design of the study and writing of the manuscript. All authors have read and approved the manuscript.

## Declaration of competing interest

T. Kishimoto has received consultant fees from Otsuka, Pfizer, and Dainippon Sumitomo, and speaker's honoraria from Banyu, Eli Lilly, Dainippon Sumitomo, Janssen, Novartis, Otsuka, and Pfizer. KF has received speaker's honoraria from Novartis and Otsuka. HT is an employee of FRONTEO. TH received speaker's honoraria from Yoishitomi. T. Kikuchi has received speaker's honoraria from Astellas, Dainippon Sumitomo, Eli Lilly, Janssen, MSD, Otsuka, Yoshitomi Yakuhin, Pfizer, and Takeda. JM has received speaker's honoraria from Eli Lilly, Janssen, Otsuka, MSD, Shionogi, and Pfizer. MM has received speaker's honoraria from Daiichi Sankyo, Dainippon-Sumitomo Pharma, Eisai, Eli Lilly, Fuji Film RI Pharma, Janssen Pharmaceutical, Mochida Pharmaceutical, MSD, Nippon Chemipher, Novartis Pharma, Ono Yakuhin, Otsuka Pharmaceutical, Pfizer, Takeda Yakuhin, Tsumura, and Yoshitomi Yakuhin. Also, he received grants from Daiichi Sankyo, Eisai, Pfizer, Shionogi, Takeda, Tanabe Mitsubishi, and Tsumura. Other authors have no conflict of interest.
